# Editorial: Emerging trends in genetic engineering of microalgae

**DOI:** 10.3389/fbioe.2024.1403711

**Published:** 2024-04-04

**Authors:** Jian Li, Spiros N. Agathos, Zhengquan Gao

**Affiliations:** ^1^ Qingdao Innovation and Development Base, Harbin Engineering University, Qingdao, China; ^2^ Earth & Life Institute, Catholic University of Louvain, Louvain-la-Neuve, Belgium; ^3^ School of Pharmacy, Binzhou Medical University, Yantai, China

**Keywords:** microalgae, genetic engineering, wastewater treatment, bioenergy, food security, sustainability, healthcare

Microalgae are a group of phylogenetically diverse microorganisms, the majority of which can perform photosynthesis. Microalgae are predominantly aquatic unicellular eukaryotes, but cyanobacteria, the photosynthetic unicellular prokaryotes, are often categorized as procaryotic microalgae due to similar physiology and biotechnological applications. In fact, cyanobacteria first acquired the ability to photosynthesize through evolution and then transferred this ability to eukaryotic microalgae through endosymbiosis, and thus procaryotic and eucaryotic microalgae are phylogenetically connected (Thoré et al., 2023). Microalgae play an important role in the evolution of the earth and its biosphere. Cyanobacteria were pioneers in the production of oxygen and the conversion of carbon dioxide into biomass, making heterotrophic and aerobic organisms possible on Earth, and to this day, microalgae may be the most important biological players in the geochemical cycles on Earth. They are the most important primary producers in aquatic ecosystems and provide food for all aquatic animals. Microalgae are a phylogenetically very diverse group of organisms that may comprise more than 70,000 species, and only a very small fraction of them have actually been isolated, identified and reported, leaving them among the least exploited biological resources on Earth (Grama et al., 2022). Exploration of microalgae for biotechnological applications could provide future solutions to global problems faced by all of us, such as environmental sustainability, food security, energy supply, healthcare and so on. Therefore, it may be worthwhile to bioprospect microalgae for strategic solutions to our global challenges due to their biodiversity, metabolic versatility and microscopic nature.

Despite this great potential, so far only a few microalgal species have been industrially exploited for niche markets such as health food or aquaculture feed applications. The primary limiting factors for the development of socially and economically attractive microalgal processes are the low surface productivity and the high cost of biomass production, compared to those from either conventional agriculture or the industrial fermentation sector. It would be highly constructive to address this challenge with the modern genetic engineering tools available for microalgae. One perspective is to engineer the light-harvesting systems of microalgae for more efficient light utilization (Hu et al., 2023). For industrial autotrophic cultivation of microalgae, light is still the limiting factor of production, and enhancing light utilization efficiency means higher photosynthetic rates and overall productivity in the same production facility. Another idea is to engineer key enzymes in the Calvin cycle for higher CO_2_ assimilation efficiency. Improving the efficiency of key enzymes such as Rubisco would facilitate the overall process of photosynthesis and again increase the area’s productivity. There are also other alternative strategies for the genetic engineering of microalgae for strain improvement, such as transcription factor engineering, but none of the above approaches has really drawn much attention from the industry so far although these concepts have been proven extensively in laboratory settings (Barati et al., 2021).

This Research Topic was intended to solicit the submission of research or review articles focusing on the genetic engineering of microalgae for industrial applications or the development of strains with industrial potential and to update the frontiers of this field of research, and ultimately we received two reviews and three research articles. The review article entitled “*Genetic engineering to enhance microalgae-based produced water treatment with emphasis on CRISPR/Cas9: A review*” (Hassanien et al.) was authored by a group of scientists from Qatar, which may be one of the most water-stressed countries in the world (Ajjur and Al-Ghamdi, 2022). The authors reviewed the research efforts in the genetic engineering of microalgae for the treatment of water produced by the oil and gas industry and elaborated on all the genetic engineering methodologies utilized to develop microalgal strains with higher bioremediation efficiency for water treatment, with an emphasis on CRISPR/Cas9 (Hassanien et al.). The other review entitled “*Advances in light system engineering across the phototrophic spectrum*” focused on recent genetic engineering efforts to modify the light systems of all photosynthetic organisms including cyanobacteria, microalgae and plants (Dennis and Posewitz). The authors comprehensively examined the experimental data and research results using similar strategies or methodologies, such as depigmentation, applied to different categories of organisms. By comparing the results of similar experimental strategies applied to different species, not only more insightful observations could be made, but also more insightful conclusions could be drawn. The research article entitled “Cas9 deletion of lutein biosynthesis in the marine microalga *Picochlorum celeri* reduces photosynthetic pigments while sustaining high biomass productivity” represents another example of using the genetic engineering strategy of depigmentation to enhance photosynthetic efficiency (Cano et al.). The authors were able to delete genes for chlorophyll and lutein synthesis in *Picochlorum celeri*, using CRISPR/Cas9 technology, and they found that transformants with the lutein synthesis gene deleted had higher light-use efficiency and could accumulate more biomass than the wild type. The research article entitled “*Unlocking microalgal host-exploring dark-growing microalgae transformation for sustainable high-value phytochemical production*” reported a case of genetic engineering of microalgae from another perspective (Jareonsin et al.). The authors were able to develop transgenic cell factories employing heterotrophic microalgae as hosts, and in the process, they transformed *Chlorella sorokiniana*, using an *Agrobacterium*-mediated system with some reporter genes. Although the level of heterologous gene expression was not quantified, the proof of concept was well demonstrated. The last research article entitled “*Screen of antibiotics to obtain axenic cell cultures of a marine microalga Chrysotila roscoffensis*” reported the sensitivity of this microalga to a number of antibiotics (Liu et al.). The sensitivity and resistance of microalgal species to certain antibiotics can constitute essential knowledge for researchers to develop selection markers for transgenic microalgae, and obtaining an axenic culture of microalgae could be the first step for further genetic engineering procedures or for heterotrophic/mixotrophic cultivation of microalgae. It was found that *Chrysotila* was able to tolerate ampicillin, kanamycin, streptomycin, gentamicin and geneticin, but it was sensitive to bleomycin, hygromycin B, paromomycin, and chloramphenicol. Although the possible reasons why the microalga was sensitive to some antibiotics and resistant to others were not discussed in the paper, it is our opinion that it can be very interesting research to investigate the correlation between microalgal lineages and the mechanism(s) of antibiotic action.
